# The C-Terminal Fragment of Agrin (CAF), a Novel Marker of Renal Function, Is Filtered by the Kidney and Reabsorbed by the Proximal Tubule

**DOI:** 10.1371/journal.pone.0157905

**Published:** 2016-07-05

**Authors:** Arezoo Daryadel, Monika Haubitz, Marta Figueiredo, Dominik Steubl, Marcel Roos, Armin Mäder, Stefan Hettwer, Carsten A. Wagner

**Affiliations:** 1 Institute of Physiology and Zurich Center for Integrative Human Physiology (ZIHP), University of Zurich, Zurich, Switzerland; 2 Neurotune AG, Schlieren, Switzerland; 3 Department of Nephrology, Klinikum rechts der Isar, Munich, Germany; Emory University, UNITED STATES

## Abstract

Agrin, a multidomain proteoglycan and neurotrypsin, a neuronal serine protease, are important for forming (neuromuscular) synapses. Proteolytical activity of neurotrypsin produces a C-terminal fragment of agrin, termed CAF, of approximately 22 kDA molecular size which also circulates in blood. The presence of CAF in urine suggests either glomerular filtration or secretion into urine. Blood levels of CAF have been identified as a potential novel marker of kidney function. Here we describe that several nephron segments in the mouse kidney express agrin and neutrotrypsin in addition to the localization of both protein in the glomerulum. Agrin mRNA and protein was detected in almost all nephron segments and mRNA abundance was highest in the inner medullary collecting duct. Neurotrypsin mRNA was mostly detected in the thick ascending limb of the loop of Henle, the distal convoluted tubule, and the inner medullary collecting duct. Moreover, we show that the proximal tubule absorbs injected recombinant CAF by a process shared with receptor-mediated and fluid phase endocytosis. Co-injection of CAF with recombinant human transferrin, a substrate of the receptor-mediated endocytic pathway as well as with FITC-labelled dextran (10 kDa), a marker of fluid phase endocytosis, showed partial colocalization of CAF with both markers. Further colocalization of CAF with the lysosomal marker cathepsin B suggested degradation of CAF by the lysosome in the proximal tubule. Thus, the murine kidney expresses agrin and neurotrypsin in nephron segments beyond the glomerulum. CAF is filtered by the glomerulum and is reabsorbed by endocytosis by the proximal tubule. Thus, impaired kidney function could impair glomerular clearance of CAF and thereby increase circulating CAF levels.

## Introduction

Agrin is a large proteoglycan of approximately 250 kDa that is highly expressed in brain and in the neuromuscular junction where it serves, among other functions, the positioning of acetylcholine receptors and synaptogenesis [1,2]. Moreover, agrin is also highly expressed in the kidney where it is the major heparan sulfate proteoglycan in the glomerular and tubular basement membrane and a ubiquitous component of the extracellular matrix [3,4,5,6].

Agrin is cleaved by neurotrypsin, a serine protease, generating a 110 kDa fragment (CAF110) by cleavage at the alpha site, whereas cleavage at the beta site produces the 22 kDa C-terminal fragment (CAF22) [7]. In human urine, CAF22 has been detected but not CAF110 [8,9]. However, it is currently unclear whether this CAF22 originates from the kidney or is filtered from blood into urine via the glomerulum. Serum CAF22 (sCAF) has recently emerged as a novel biomarker for kidney function and correlates with other markers such as eGFR in septic patients, renal transplant recipients, patients with peritoneal dialysis, and patients with diabetic nephropathy [8,10,11,12,13,14]. In patients with impaired kidney function, CAF22 levels are elevated and highly correlate with loss of function and functional recovery. Thus, CAF22 may serve as a new marker of kidney function that responds even faster to acute changes in organ function than other traditionally used markers. However, the mechanisms underlying the dependence of serum CAF22 levels on kidney function have not been examined.

Small proteins are partly filtered in the renal glomerulum and can be either found in urine or are mostly reabsorbed by various endocytic mechanisms including receptor-mediated endocytosis and fluid-phase endocytosis. Both processes mainly occur in the proximal tubule. Receptor-mediated endocytosis involves the endocytic receptors megalin and cubilin binding a large variety of low molecular weight proteins including transferrin or cathepsin B [15,16,17,18]. Proteins endocytosed by this pathway are delivered via early and late endosomes to the lysosome [18]. Alternatively, substrates may be endocytosed by fluid-phase endocytosis [19]. Horseradish peroxidase or low molecular weight dextrans are classic markers for this pathway [19,20].

Thus, we aimed to examine whether the murine kidney expresses neurotrypsin and produces the small 22 kDa agrin fragment (CAF) and could thereby contribute to circulating or urinary CAF22 levels. Second, we investigated whether CAF22 could be filtered by the glomerulum and reabsorbed by the proximal tubule. Our results demonstrate that agrin and neurotrypsin are both present in murine kidney and that their mRNA expression profile along the nephron partly overlaps. Agrin protein is detected, as expected, in the glomerulum and in the basement membrane of all nephron segments and is paralleled by strong staining for the CAF22 fragment produced by neurotrypsin as indicated by its absence from the kidneys of neurotrpysin KO mice. Moreover, we provide evidence that injected CAF22 accumulates in the endocytic pathway of the proximal tubule and overlaps highly with markers of receptor-mediated endocytosis suggesting that CAF22 may be filtered in the glomerulum, reabsorbed by the proximal tubule via receptor-mediated endocytosis and degraded in lysosomes.

## Methods

### Animal experiments

Experiments were performed in 8–10 weeks old male mice with 25–30 g body weight. C57B1/6J mice (WT) purchased from Janvier Labs (France) and neurotrypsin knock out (NT^-/-^) were used. The generation and genotyping of neurotrypsin KO mice has been previously described [21]. All animal experiments were conducted according to Swiss laws for the welfare of animals and were approved by the Zurich Veterinary Authority. The animals had free access to food and tap water.

### Human Recombinant CAF (hCAF) injection into mice

Mice were pretreated 60 min. before experiments with an injection of leupeptin (0.5 mg/mouse, Sigma Aldrich, Buchs, Switzerland), an inhibitor of lysosomal degradation. Wildtype and neurotrypsin KO mice were anesthetized by i.p. injection of xylazin and ketamine and were injected intravenously with 100 μl of a mixture containing recombinant hCAF22 (100 ng/mouse, produced by Neurotune) dissolved in 0.9% NaCl and FITC-labeled sinistrin (100 ng/ mouse). FITC-sinistrin is cleared from circulation exclusively by glomerular filtration and served as control for successful injection. Before injection of hCAF, the urinary bladder was completely emptied through a small abdominal incision to allow collection of urine produced during the experimental period. Mice received also a bolus i.p. of 0.5 ml of 25 mM NaHCO_3_/125 mM NaCl to prevent dehydration and to promote diuresis. Mice were kept warm at 37°C by placing animals on a heating tablet for the rest of the experiment. Twenty or 60 minutes after hCAF injections, urine was collected from the bladder, heparinized blood collected from the vena cava, and mice perfused with 3% PFA/PBS through the heart to obtain fixed kidneys for later immunohistochemistry and localization of recombinant hCAF.

### Dissection of mouse nephron segments

Mice were anesthetized and perfused through the left heart ventricle with 15 ml of a HBSS—Hank's Balanced Salt Solution 1x (Gibco) containing 1 mg/ml collagenase type IA (Sigma C9891). Kidneys were immediately removed, the capsule removed, and thin coronal slices containing both cortex and medulla were prepared. The inner medulla was removed. Tissue was incubated at 37°C in a water bath for approximately 15 min without shaking in a HBSS solution containing 1 mg/ml collagenase type IA (Sigma C9891). When the medium became cloudy upon gentle shaking, the digestion was stopped by transferring the tubules to ice, carefully removing the supernatant and washing twice with 10 ml ice-cold HBSS to remove collagenase. Dissection was performed under a stereo microscope at 4°C using two fine forceps (Dumont no. 5). Approximately 50 segments per preparation were collected and placed in 300 μl RLT-Buffer (Qiagen, Basel, Switzerland) containing 3 μl of 2-β-mercaptoethanol. Segments were dissected separately from five animals. Collected tissue was immediately frozen at –80°C until mRNA extraction.

### RNA extraction and qPCR

To determine Agrin and neurotrypsin mRNA abundance in mouse different organs such as kidney, lung, fat, brain, long and small intestine, heart muscle, the organs were homogenized with RLT buffer added with betamercaptoethanol from RNeasy Micro Kit (Qiagen) and RNA was extracted according to the manufacturer's instructions. RNA was bound to columns and treated with DNase for 15 min at room temperature to reduce genomic DNA contamination. Quality and concentration of the isolated RNA preparations were analyzed using the ND-1000 spectrophotometer (NanoDrop Technologies). Total RNA samples were stored at –80°C.

To generate complementary DNA (cDNA), each RNA sample was diluted to 10 ng/μl and was used as a template for reverse transcription using the TaqMan Reverse Transcription Kit (Applied Biosystems, Forster City, CA). Quantitative real-time (qRT-PCR) was performed on the ABI PRISM 7700 Sequence Detection System (Applied Biosystems). Primers for all genes of interest were designed using Primer Express from Applied Biosystems (S1 Table). Probes were labeled with the reporter dye FAM at the 5' end and the quencher dye TAMRA at the 3' end (Microsynth, Balgach, Switzerland). The specificity of all primers was first tested in a standard PCR and always resulted in a single product of the expected size on 1.5% agarose gels (data not shown). Real-time PCR reactions were performed using the TaqMan Universal PCR Master Mix (Applied Biosystems). Briefly 3 μl cDNA, 0.8 μl of each primer (25 μM), 0.4 μl labeled probe (5 μM), 5.2 μl RNase-free water, 10 μl TaqMan Universal PCR Master Mix reached 20.2 μl of final reaction volume. Reaction conditions were: denaturation at 95°C for 10 min followed by 50 cycles of denaturation at 95°C for 15 s and annealing/elongation at 60°C for 60 s with autoramp time. All reactions were run in duplicate. To analyze the data, we set the threshold to 0.06 as this value had been determined to be in the linear range of the amplification curves for all mRNAs in all experimental runs. The expression of gene of interest was calculated in relation to hypoxanthine guanine phosphoribosyl transferase (HPRT). Relative expression ratios were calculated as R = 2^[Ct(HPRT)–Ct(test gene)]^, where Ct represents the cycle number at the threshold 0.06.

### Immunoblotting

Determination of recombinant CAF22 from mouse body fluids by Western blot analysis was performed as described in Hettwer et al., 2013 [22]. In brief, urine was diluted 20 times in 1 × Lämmli buffer and heated for 5 min to 95°C. Samples (10 μl) were loaded onto 4–12% NUPAGE gels (Inivitrogen). Separated proteins were transferred to PVDF (Invitrogen) membranes by wet blotting for 60 min at 24 V. CAF containing fragments in human serum were detected using the biotinylated monoclonal anti-CAF antibody 28A6H11 [22]. This mouse monoclonal antibody is directed against human CAF2 and the binding epitope was determined by epitope mapping to be “TFVE” close to the C-terminus of the CAF22 fragment.

As reporter molecule, streptavidin-poly-HRP conjugate (PIERCE) was used. For detection, Chem Glow West substrate (Alpha Innotech) was applied and the chemiluminescence was recorded with a Stella imaging system (Raytest, Germany).

### Preparation of CAF

CAF22 variants were produced and purified as described [23] using constructs which allowed cleaving off the N-terminal His-tag using the prescission cleavage site directly after the tag. After purification via IMAC, the tag was removed by prescission cleavage. Human recombinant CAF22 shares about 91% identity at the amino acid level with mouse CAF22.

### Immunohistochemistry

C57B1/6J mice were anesthetized with ketamine: xylazin and perfused through the left ventricle with phosphate-buffered saline (PBS) followed by paraformaldehyde-lysine-periodate (PLP) fixative [24]. Kidneys were removed and fixed overnight at 4°C by immersion in PLP. Kidneys were washed 3 times with PBS and 5 μm cryosections were cut after cryoprotection with 2.3 M sucrose in PBS for at least 12 h. Immunostaining was carried out as described previously [25]. Briefly, sections were shortly incubated in microwave with Tris-HCL [pH 10], following 1% (wt/vol) SDS for 5 min for retrieval of antigenic sites, washed 3 times with PBS and incubated with PBS containing 1% bovine serum albumin for 15 min prior to the primary antibody. The primary antibodies mouse monoclonal anti cleaved Agrin Abs (CAF; 14B7B8; 1:1000) and (CAF; 12A11D11; 1:1000) (provided by Neurotune), rabbit polyclonal anti Agrin (serum 204 [26], provided by Markus A. Rüegg, Basel, Switzerland) 1:2000 were diluted in PBS and applied overnight at 4°C. Sections were then washed twice for 5 min with high NaCl PBS (PBS + 18 g NaCl/l), once with PBS, and incubated with dilutions of the secondary antibodies (donkey anti-rabbit 594 (1:500), donkey anti-mouse 488 (1:200), donkey anti-mouse Alexa 594 (1:500), donkey anti-rabbit Alexa 488 (1:500) (Molecular Probes, Oregon, USA) and from DAPI (1 mg/ml) (1:500) for 1 h at room temperature. Sections were again washed twice with high NaCl PBS and once with PBS before mounting with VectaMount (Vector Laboratories, Burlingame, CA). Sections were viewed with a confocal laser scanning microscope (Zeiss LSM 700, Carl Zeiss) or a Leica DFC490 charged-coupled device camera attached to a Leica DM 6000 fluorescence microscope (Leica, Wetzlar, Germany). Confocal microscope pinhole was set at 1 Airy unit and pixel size at 90nm and a 40×/1.3 oil DIC M27 objective was used. Images were processed (overlays) using Adobe Photoshop and ImageJ software (http://rsb.info.nih.gov/ij/).

## Results

### Expression of agrin and neurotrypsin in mouse kidney

**Fig 1:**
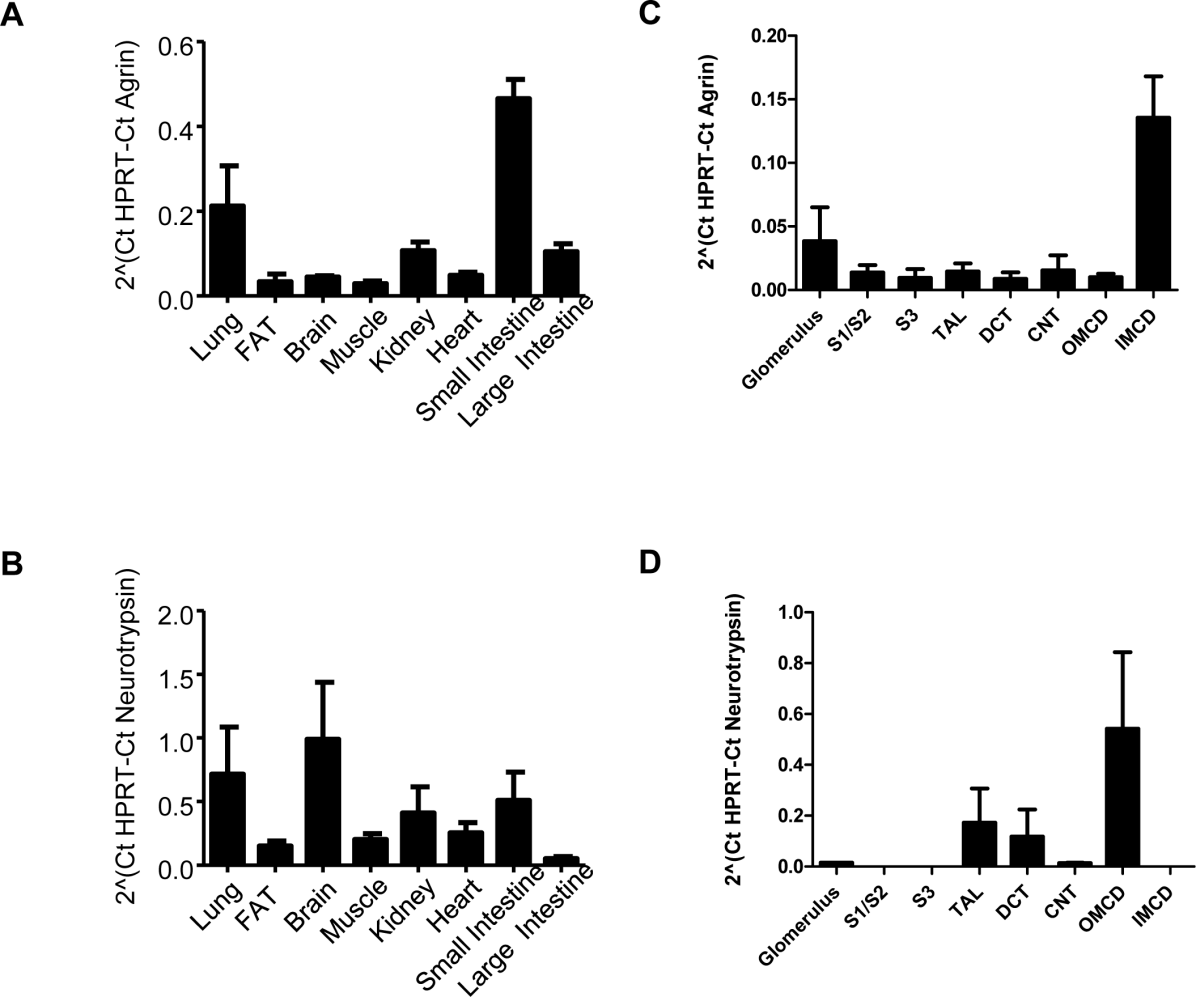
mRNA expression of agrin and neurotrypsin. **(A,B)** mRNA was extracted from various mouse organs and relative mRNA abundance of agrin and neurotrypsin assessed by real-time RT-PCR (n = 4 animals). **(C,D)** Mouse nephron segments were isolated by hand-dissection and relative mRNA abundance of agrin and neurotrypsin measured by real-time RT-PCR (n = 4 preparations per nephron segment).

Expression of agrin and neurotrypsin mRNA was tested in various in mouse organs by semi-quantitative RT-PCR (Fig 1A and 1B). mRNA of both transcripts was detected in lung, brain, small and large intestine, white adipose tissue, heart, kidney, and skeletal muscle. However, relative expression levels differed between organs and between agrin and neurotrypsin. The highest levels for agrin mRNA were found in lung and small intestine followed by kidney and large intestine. In contrast, the highest mRNA abundance for neurotrypsin mRNA was detected in lung and brain followed by kidney and small intestine.

We examined further the relative mRNA expression of agrin and neurotrypsin in hand-dissected mouse nephron segments. Agrin mRNA was detected in the glomerulum, and all nephron segments. The highest levels of agrin mRNA were found in the inner medullary collecting duct (Fig 1C). In contrast, neurotrypsin mRNA was only detected in the glomerulum, the thick ascending limb of the loop of Henle (TAL), the distal convoluted tubule (DCT) and the inner medullary collecting duct (Fig 1D).

**Fig 2:**
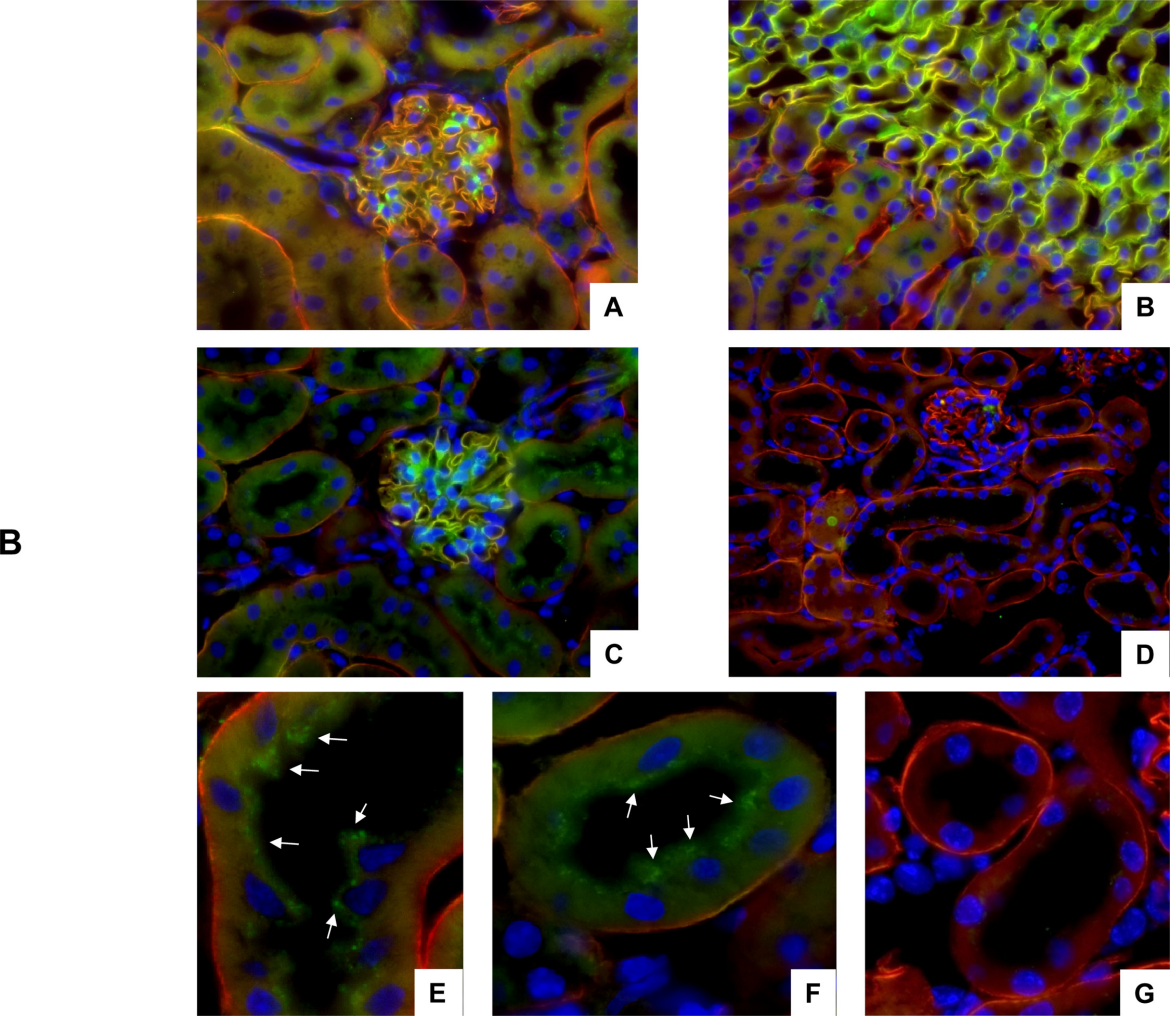
Detection of full length agrin and CAF22 in mouse kidney. Kidneys from wildtype and NT^-/-^ mice were stained with antibodies against full length agrin (red) and CAF (green). Cell nuclei were marked with DAPI (blue). (**A,B,C**) Localization of full length agrin and CAF22 in the renal cortex (A,C) and at the transition between outer and inner medulla in wildtype kidney. (**D**) No staining related to CAF22 was detected in kidneys from NT^-/-^ mice. Original magnification 400x. (**E,F**) Higher magnification of proximal tubules in wildtype kidney show a punctuate subapical staining for CAF22 (arrows). (**G**) No subapical staining in proximal tubules of kidneys from NT^-/-^ mice.

The expression of agrin protein in mouse kidney was further tested by immunohistochemistry (Fig 2). Immunohistochemistry showed agrin expression in the glomerulum and in the basement membrane around tubules in all areas of the kidney confirming previous reports demonstrating that agrin is a part of basement membranes in the glomerulum and kidney tubules [3,4,5,6]. Next, we also tested the occurance of the 22kDa C-terminal agrin fragment CAF by immunohistochemistry (Fig 2). Using the 14B7B8 anti-CAF22 antibody we detected clear staining In kidneys from wildtype mice but not in kidneys from NT^-/-^ that partly colocalized with agrin in the basement membrane of the glomerulum and the renal tubules (Fig 2). Thus, this antibody recognizes only CAF22 but not full length agrin. It was further used for determination of CAF22 handling by the kidney as described below.

### CAF22 is filtered into urine

**Fig 3:**
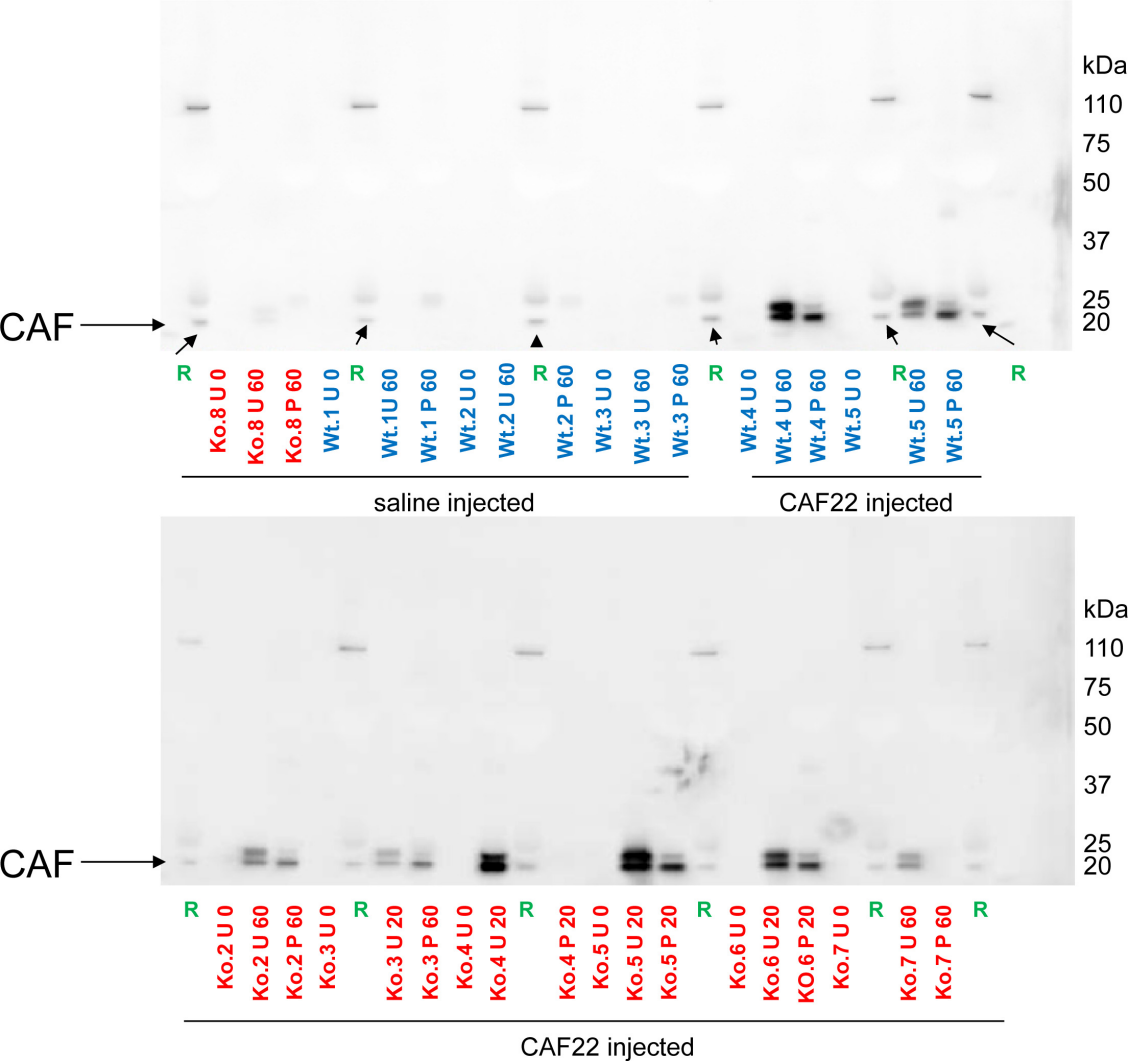
Presence of CAF22 in plasma and urine of CAF22 injected mice. Wildtype (wt) and NT^-/-^ (ko) mice were injected with saline or human recombinant CAF22. Urine was collected before injection (U 0), 20 min (U 20) or 60 min (U 60) after injection by puncture of the urinary bladder. Plasma was collected at the end of experiments after 20 min (P 20) or 60 min (P 60). Human plasma was used as positive control (R). The arrow indicates the expected size for CAF22. Typical examples from the different treatments are shown.

Some CAF22 related staining was also detected in large dots in the subapical compartment of proximal tubules of WT mice (Fig 2E and 2F). This staining was absent from NT^-/-^ kidneys (Fig 3G). This staining together with the small molecular size of CAF22 suggested that CAF may be filtered in the glomerulum or derive from the basement membrane of the glomerulum and may then be subsequently reabsorbed in the proximal tubule by endocytosis. To test this hypothesis, we injected human recombinant CAF22 into NT^-/-^ KO mice since all CAF22 that would be detected in urine or in the proximal tubule would likely reach these sites by glomerular filtration. To rule out differences in glomerular handling of CAF22 in NT^-/-^ mice, also some wildtype mice were injected with human recombinant CAF22. To further facilitate detection of this CAF22 in the endocytic and lysosomal compartment, mice were pretreated 1hr prior to the CAF22 injection with leupepetin (0.5 mg/mouse) to block lysosomal degradation. Plasma, urine and kidneys were collected 20 or 60 min after CAF injection. Immunoblotting of plasma samples for CAF22 readily detected CAF22 confirming successful injection into the vascular system (Fig 3). Moreover, CAF22 was also detected in urine samples collected from the bladder of CAF22 injected mice (Fig 3).

### CAF22 is reabsorbed by the proximal tubule via endocytosis

**Fig 4:**
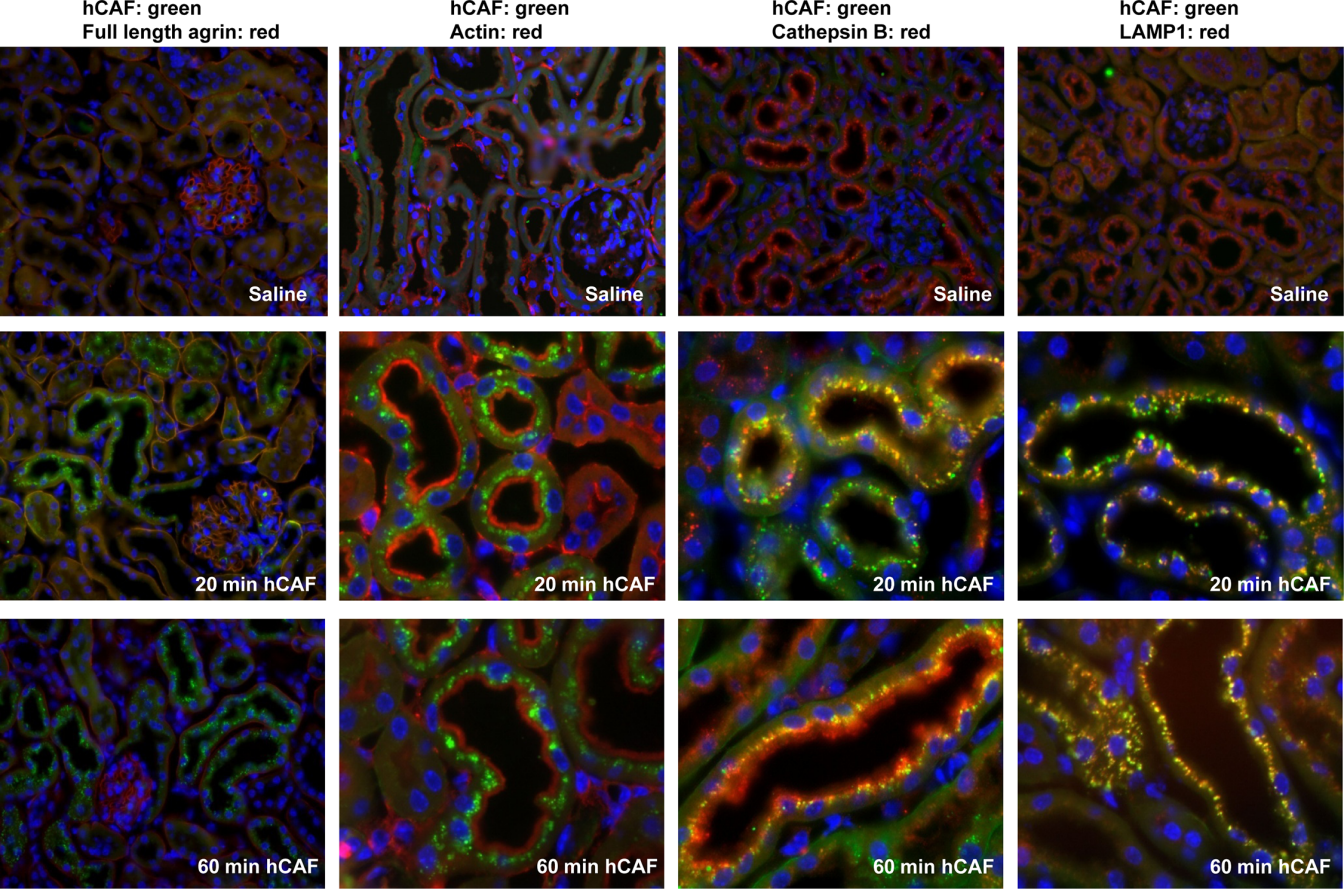
Detection of human recombinant CAF22 in kidney. NT^-/-^ mice were pretreated with leupeptin and injected 60 min later with saline or human recombinant CAF22. Mice were fixed by perfusion 20 or 60 min after saline or CAF22 injection and tissue sections stained for CAF22 (green), actin (red), cathepsin B (red) or LAMP1 (red). Cell nuclei were marked with DAPI (blue). Original magnification 400–1000 x.

**Fig 5:**
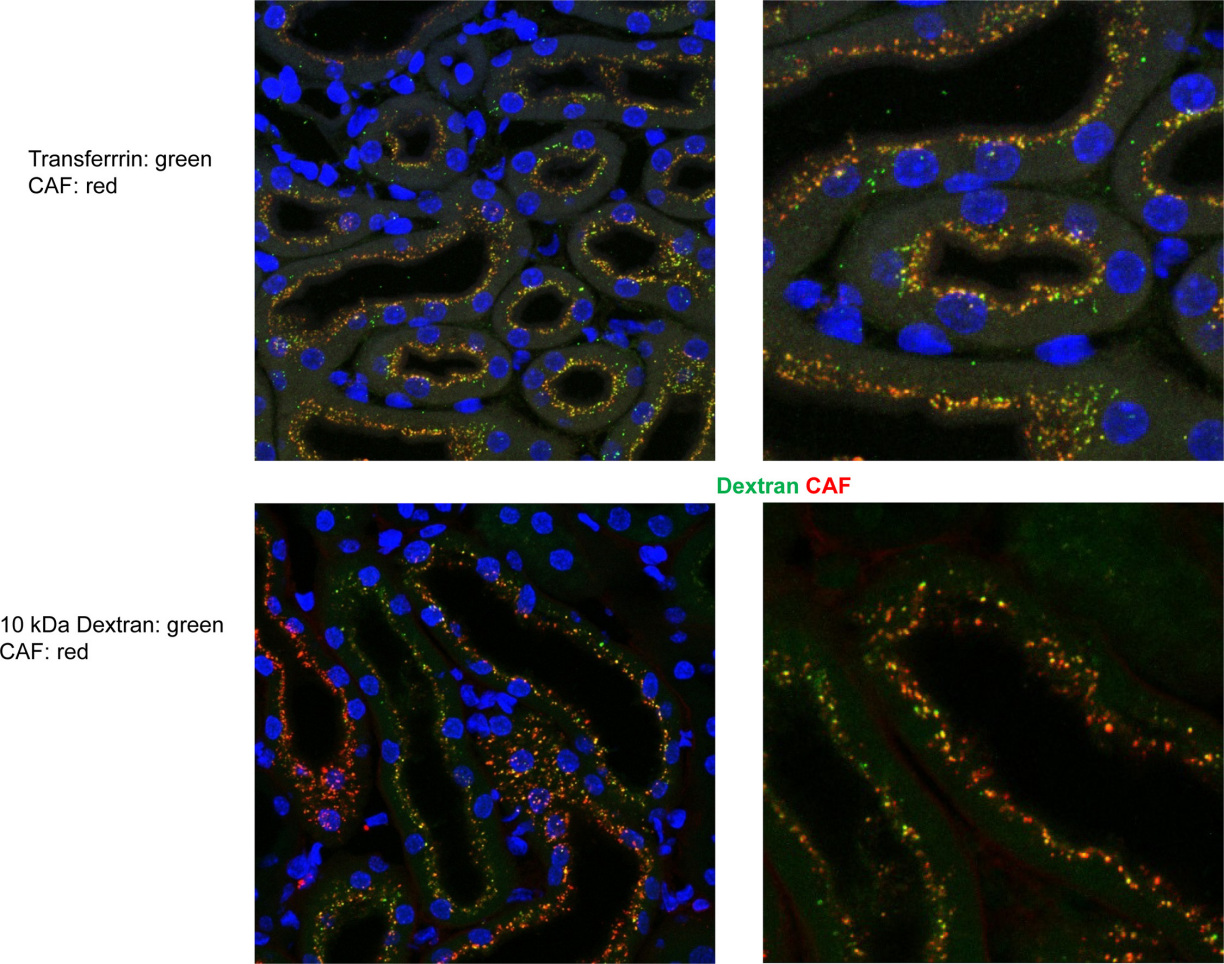
Colocalization of human recombinant CAF22 with markers of fluid-phase and receptor-mediated endocytosis. NT^-/-^ mice were pretreated with leupeptin and injected 60 min later with human recombinant CAF22 in combination with either human recombinant transferrin or 10 kDa FITC-dextran. Mice were fixed by perfusion 10 min after injection and tissue sections stained for CAF22 (red) or human recombinant transferrin (green). FITC-dextran was detected as green staining. Cell nuclei were marked with DAPI (blue). Original magnification 1000 x.

Immunohistochemistry was used to localize CAF22 in kidneys from NT^-/-^ injected either with saline or CAF22, 20 or 60 min after CAF22 injections. CAF related staining was detected in a strong punctuate pattern in the subapical compartment of proximal tubule in NT^-/-^ mice injected CAF22 but not with saline. Staining the apical brush border membrane with antibodies for actin, we saw that CAF22 staining was in smaller vesicles near the brush border membrane 20 min after injection. One hour after CAF22 injection, CAF22 staining accumulated in larger vesicles that appeared to be localized further away from the brush border membrane. To further characterize the structures containing CAF22, we used antibodies against cathepsin B, a lysosomal enzyme that is taken up from urine via receptor-mediated endocytosis [27]. Twenty minutes after CAF22 injection, CAF22 and cathepsin B strongly colocalized in subapical vesicles, likely reflecting clathrin-coated and/or early endosomal vesicles (Fig 4)[27]. One hour after CAF22 injection, CAF22 overlapped with cathepsin B staining in vesicles. However, cathepsin B staining at the brush border membrane and in the subapical compartment did not overlap with CAF22 likely due to ongoing retrieval of endogenous cathepsin B. Colocalization of CAF22 with the lysosomal marker LAMP1 showed a higher degree of overlap after 60 minutes than after 20 minutes (Fig 4) suggesting that CAF22 had not fully reached the lysosomal compartment at the earlier time point. These data suggested that CAF22 is mostly reabsorbed from urine and internalized by the same route as receptor-mediated endocytosis. To further test this possibility, human recombinant CAF22 was coinjected with human recombinant transferrin, a prototypical substrate for the receptor-mediated endocytosis pathway [15]. Moreover, we also assessed the possibility of CAF22 reabsorption by the route of fluid-phase endocytosis by injecting 10 kDa FITC-labeled dextran, a marker for this pathway [20]. Human recombinant transferrin was detected with an antibody detecting human but not endogenous mouse transferrin. In saline injected mice, no transferrin and CAF22 related signal was detected (data not shown) whereas a high degree of overlap between CAF22 and transferrin immunosignals was detected in kidneys from coinjected mice (Fig 5). Some overlap was also detected between CAF22 and 10 kDa FITC-dextran signals was seen. However, this overlap appeared to be less strong as indicated by many vesicles only positive for CAF22 but not FITC-dextran (Fig 5).

## Discussion

Our study confirms the expression and localization of agrin in basement membranes of the renal glomerulum and different nephron segments [3,5,6,28,29,30]. It also demonstrates that neurotrypsin, a protease cleaving agrin, is expressed in various nephron segments. Unfortunately, the lack of suitable antibodies prohibited a more detailed localization of neurotrypsin protein in mouse kidney. All antibodies tested stained kidneys from wildtype and neurotrypsin KO mice similarly. However, we provide indirect evidence of the proteolytic activity of neurotrypsin in mouse kidney by staining for the 22 kDa C-terminal agrin fragment (CAF22) that is produced by neurotrypsin-dependent agrin cleavage. CAF22 was readily detectable in wildtype kidney but not in kidneys from neurotrypsin KO mice proofing that the used antibody detects only liberated CAF22 but not the intact C-terminus of full length agrin or CAF110. The epitope detected by the 14B7B8 antibodv has been mapped to the amino acid sequence FVEYL close to the C-terminus of the cleaved agrin fragment. We assume that there is a sterical hindrance of non-cleaved agrin which prevents the antibody from accessing the epitope. Only the free CAF C-terminus by neurotrypsin cleavage renders the conformation of agrin so that this special antibody can detect cleaved agrin fragments. CAF22 related staining was visible in all areas of the kidney that expressed also agrin suggesting that a part of agrin undergoes constant cleavage by neurotrypsin. Agrin cleavage occurs in other tissues only in the presence of neurotrypsin and neurotrypsin is up to date the only known protease mediating agrin cleavage in vivo [23,31]. The biological relevance of this cleavage in kidney is currently unknown.

More importantly, our data support the hypothesis that CAF22 circulating in blood is cleared, at least in part, from circulation by glomerular filtration. We injected recombinant human CAF22 i.v. into NT^-/-^ mice that lack endogenous production of CAF22 and could detect human CAF22 both in urine and accumulated in subapical vesicles along the proximal tubule. In theory, CAF22 could have reached the urine from the bloodstream by glomerular filtration or active secretion by the proximal tubule, or a combination of both. However, the molecular size of CAF22 is sufficiently small to be filtered across the glomerular barrier like many other small proteins appearing in primary urine including cathepsins or transferrin [15,16,27]. Moreover, the immunolocalization of CAF22 in native wildtype animals or NT^-/-^ animals injected with recombinant CAF22 showed strong accumulation of CAF22 in subapical compartments but we never detected any CAF22 related staining at the basolateral pole of cells suggesting that secretion (or transcytosis) from the basolateral to the apical/luminal side of cells is unlikely to occur. We cannot entirely rule out that human recombinant CAF22 might behave differently from endogenous murine CAF22. However, the fact that endogenous CAF22 was detected in a similar subapical localization as the injected human recombinant CAF22 suggests similar handling by the kidney.

Staining of CAF22 in proximal tubules is reminiscent of other low molecular weight proteins that are reabsorbed from urine by the proximal tubule [15,16,27]. Thus, we tested whether CAF22 would share pathways of internalization with other proteins known to be localized in or internalized by receptor-mediated endocytosis. Indeed, a high degree of overlap was detected with cathepsin B, an enzyme that is absorbed from urine via the endocytic receptors megalin and cubilin and targeted to lysosomes. Also, LAMP1, a protein highly expressed in late endosomes and lysosomes colocalized with CAF22. Since receptor-mediated endocytosis and fluid phase endocytosis eventually converge at the level of the late endosomes and lysosomes, we further tested two specific substrates of receptor-mediated endocytosis, transferrin, and fluid phase mediated endocytosis, the low molecular weight dextran FITC-dextran (10 kDa). Both substrates showed a partial overlap with CAF22 when coinjected suggesting that CAF22 may take both routes to be internalized and routed to the lysosome. Even though difficult to quantify, it appeared that the overlap might be stronger for transferrin/CAF than for FITC-dextran/CAF.

In summary, we provide evidence that CAF22 is cleared from circulation by glomerular filtration. Filtered CAF22 may then be reabsorbed by the proximal tubule by receptor-mediated and fluid-phase mediated endocytosis and targeted to lysosomes for degradation. These findings may imply that impaired renal function may rapidly increase circulating CAF22 levels and thereby facilitate the detection of reduced kidney function by elevated CAF22 serum levels. It may further suggest that increased urinary CAF22 levels may be another biomarker for reduced proximal tubular function similar to other low molecular weight proteins such as β2 microglobulin. However, this is due to further examinations.

## Supporting Information

S1 Table Primer and probe sequences. Primers and probes used for semi-quantitative RT-PCR.
(DOC)
