# Supplementary material for: The C-Terminal Fragment of Agrin (CAF), a Novel Marker of Renal Function, Is Filtered by the Kidney and Reabsorbed by the Proximal Tubule
Source: PLoS One. 2016 Jul 5;11(7):e0157905. doi: 10.1371/journal.pone.0157905 (PMC4933355; doi:10.1371/journal.pone.0157905)
Supplement: S1 Table — Primers and probes used for semi-quantitative RT-PCR. (DOC) [file pone.0157905.s001.doc]

**Supplementary table 1**: Primer and probe sequences

|  | **Primer** | **Fw** | **Probe** | **Amplicon length** |
| --- | --- | --- | --- | --- |
|  |  | **Rv** |  |  |
| Agrin | 5' GCC TGC TTC TCC AGA AAG TG T 3' | | 5' TCT GGT CAA TGC CAG ACT CGA GAC 3' | 145 |
|  | 5' CTG TAA GCC CCA TCA CAG GT 3' | |  |  |
| Neurotrypsin | 5' AGA GAG AGG CCA CAG AAA ACA G 3' | | 5' AAC AGG ATG GGG AGA CAC AGG TCG T 3' | 113 |
|  | 5' CTC TTG GGT AAC AGA GGC ACA 3' | |  |  |
| HPRT | 5' TTA TCA GAC TGA AGA GCT ACT GTA ATG ATC 3' | | 5' TGA GAG ATC ATC TCC ACC AAT AAC TTT TAT GTC CC 3' | 127 |
|  | 5' TTA CCA GTG TCA ATT ATA TCT TCA ACA ATC 3' | |  |  |
